# Utilization of emergency and hospital services among individuals in substance abuse treatment

**DOI:** 10.1186/1747-597X-9-16

**Published:** 2014-04-03

**Authors:** Julie A Cederbaum, Erick G Guerrero, Keyon R Mitchell, Tina Kim

**Affiliations:** 1 School of Social Work, University of Southern California, 669 West 34th Street, Los Angeles, CA 90089, USA; 2 Los Angeles County Department of Public Health, Substance Abuse Prevention and Control, 1000 South Fremont Avenue, Building A-9 East, Alhambra, CA 91803, USA

**Keywords:** Emergency services, Hospital services, Substance abuse treatment

## Abstract

**Background:**

To examine risk factors for use of hospital services among racial and ethnic minority clients in publicly funded substance abuse treatment in Los Angeles County, California. We explored cross-sectional annual data (2006 to 2009) from the Los Angeles County Participant Reporting System for adult participants (*n* = 73,251) who received services from treatment programs (*n* = 231).

**Methods:**

This retrospective analysis of county admission data relied on hierarchical linear negative binomial regression models to explore number of hospital visits, accounting for clients nested in programs. Client data were collected during personal interviews at admission.

**Findings:**

Our findings support previous work that noted increased use of emergency rooms among individuals suffering from mental health- and substance use-related issues and extend the knowledge base by highlighting other important features such as treatment need, i.e., residential compared to outpatient treatment.

**Conclusions:**

These findings have implications for health care policy in terms of the need to increase prevention services and reduce costly hospitalization for a population at significant risk of co-occurring mental and physical disorders.

## Background

In the United States, the overuse of emergency room (ER) care remains a critical issue [1]. As ER visits rise, overall hospital costs increase, influencing quality of care [2,3]. Nonurgent use of the ER is problematic; of the 136 million ER visits in 2009, 4.6 million (45.1%) were attributed to drug misuse or abuse [4]. ER use by substance users also taxes the hospital system. In 2007, 41% of mental health or substance abuse patients who visited the ER were hospitalized [3], occupying needed beds and contributing to increased hospital costs [5]. Given the number of ER visits and hospitalizations attributable to substance use, understanding ways in which community-based substance use treatment facilities may reduce hospital costs is imperative.

### Hospital and ER use by individuals with substance abuse issues

Fuda and Immekus [6] determined the following common characteristics of substance abusers: they are sicker, tend to use more health services, and have higher rates of mental illness and substance abuse disorders. These individuals frequent the emergency room due to chest pain, drug-induced psychosis, depression, overdose, vehicle accidents, or drug-seeking behavior [7,8]. ER users are more socioeconomically disadvantaged and use drugs more often than non-ER users [9]. These patients also visit mental health clinics and primary care practitioners less often and frequently receive these services outside mental health and substance abuse facilities [10]. Therefore, we sought to explore the characteristics of individuals in substance use treatment most at risk of using hospital services with the goal of informing policies to improve screening and treatment protocols in substance use treatment.

## Methods

This study analyzed a subset of data collected via the Los Angeles County Participant Reporting System. This systemwide evaluation database includes information from all publicly funded substance abuse treatment programs in the county and captures treatment experiences and immediate outcomes. Client admission data were collected during personal interviews at intake. The collection form includes 10 items from the Addiction Severity Index [11] and the Drug Abuse Reporting Program [12]. These scales have been shown to be reliable measures of substance abuse severity [13], particularly among diverse populations [14], allowing for assessment of client reports from intake to discharge.

### Sample

This study included 73,251 unique patient treatment episodes between January 1, 2006, and December 31, 2009. This sample reported an average of 1.6 treatment episodes between 2006 and 2009. Of these patients, 17,362 were Black, 29,221 were Hispanic, and 26,668 were White (see Table 1). Data are presented by race/ethnicity because statistically significant mean differences between groups emerged in all categories; this may be attributable in part to the study sample size and thus differences are not reported in the text.

**Table 1:** Client characteristics by race and ethnicity using 2006–2009 data

	**Black**	**Latino**	**Non-latino white**
	***n*** **= 17,362**	***n*** **= 29,221**	***n*** **= 26,668**
	***M *** **( *SD *) or %**	***M *** **( *SD *) or %**	***M *** **( *SD *) or %**
Emergency room visits	0.16 (1.28)	0.15 (1.36)	0.20 (1.27)
Days in hospital	0.18 (1.49)	0.12 (1.16)	0.24 (1.65)
Age	39.8 (12.8)	32.2 (11.9)	37.4 (11.9)
Male	64.8	69.8	65.2
Education level	11.3 (2.7)	10.6 (2.6)	11.9 (2.6)
Homeless	29.5	20.0	28.6
Diagnosed with mental disorder	24.7	12.9	27.7
Days of mental health counseling	0.18 (1.66)	0.15 (1.70)	0.18 (1.70)
Days of psychiatric care	0.19 (1.80)	0.10 (1.21)	0.24 (1.93)
Days with physical health problems	1.69 (6.29)	1.01 (4.88)	1.60 (6.00)
Age at first drug use	21.0 (8.8)	19.0 (7.4)	19.8 (8.3)
Days of primary drug use	9.5 (11.7)	11.2 (12.7)	15.5 (13.0)
Primary drug problem			
Alcohol	19.9	17.6	26.1
Cocaine	46.3	9.5	7.6
Heroin	6.6	19.8	25.7
Marijuana	19.0	16.5	6.0
Methamphetamine	4.4	33.5	26.6
Other	3.7	3.1	8.1
Days of secondary drug use	6.9 (10.2)	6.8 (10.4)	9.6 (11.9)
Days of alcohol use	1.94 (5.99)	1.97 (6.05)	2.47 (6.91)
Children younger than 18	0.33 (0.71)	0.51 (0.86)	0.23 (0.57)
Program modality			
Outpatient	55.6	55.7	31.6
Methadone	3.2	3.4	5.5
Residential	41.3	41.0	63.0

### Dependent variables

Number of emergency room visits was measured at intake by asking, “How many times have you visited an emergency room in the past 30 days for physical health problems?” Hospitalization was measured by asking, “How many days have stayed overnight in a hospital for physical health problems in the last 30 days?”

### Independent variables

Independent variables included demographics, mental and physical health, substance use, and treatment modality. Demographic variables were (1) race and ethnicity (Black, Hispanic, or White), (2) age, (3) sex, (4) level of education, (5) homelessness, and (6) children (measured as number of children younger than 17 and younger than 5).

Mental health-related questions were: (1) “Have you ever been diagnosed with a mental illness?” (2) “How many times in the past 30 days have you received outpatient emergency services for mental health needs?” (3) “How many days in the past 30 days have you stayed for more than 24 hours in a hospital or psychiatric facility for mental health needs?” Physical health was assessed by asking, “How many days have you experienced health problems in the past 30 days?”

The five substance use questions were: (1) “What is your primary drug or alcohol problem?” (alcohol, cocaine, heroin, marijuana, or methamphetamine); (2) “How many days in the past 30 days you have used your primary drug?” (3) “What was your age at first use of your primary drug?” (4) “During the last 30 days, did you use alcohol?” (5) “How many days in the past 30 days have you used a secondary drug?”

Individuals identified as participating in either (1) outpatient treatment services; (2) a residential treatment program; or (3) a narcotic (methadone) treatment program.

### Data analysis

To test the association between explanatory variables and number of emergency room visits and days in the hospital we utilized Stata for multilevel negative binomial regression analyses, using NBREG with a log link function [15]. Negative binomial regression with robust standard errors was used to analyze overdispersed measures of ER visits and days of hospitalization, i.e., their variance was much greater than their mean [16]. Compared to Poisson regression, which is generally used to model count data, negative binomial analysis is more efficient at modeling overdispersed outcomes using the extra parameter of exposure to an event [16,17]. The CLUSTER option was used to account for the multilevel structure of the data (clients nested in programs) and to obtain more accurate estimates of standard errors [18], as suggested in other research [19]. Missing data on selected variables was less than 3% and was addressed using maximum likelihood specification in the multilevel model, as suggested by experts in missing data [20].

## Results

Our data revealed demographic, health, substance use, and program factors were associated with both ER use and hospitalization. Factors associated with ER use are presented, followed by those related to inpatient hospitalization.

### Correlates of emergency room visits

Several demographic and drug and mental health issues including race/ethnicity, sex, recent drug use, and an absence of mental health issues were associated with ER visits (Table 2). Several demographic factors were significantly associated with increased ER visits. Non-Hispanic Whites visited ERs more frequently compared to Blacks and Hispanics. Women tended to report more ER visits compared to men. Clients reporting less education, more children under the age of 18, and homelessness were also associated with more ER visits.

**Table 2:** Random effect negative binomial regression on ER visits

	**IRR**	***SE***	***p***	**95% CI**
Race/ethnicity				
Non-Latino White^a^				
Black	0.852	0.031	< .001	0.793, 0.915
Latino	0.826	0.025	< .001	0.778, 0.876
Age	1.000	0.001	.836	0.997, 1.002
Male	0.777	0.021	< .001	0.737, 0.819
Education	1.017	0.005	.001	1.007, 1.028
Homeless	1.212	0.035	< .001	1.145, 1.283
History of mental health issues	0.661	0.018	< .001	0.627, 0.698
Days of mental health counseling	1.021	0.003	< .001	1.015, 1.028
Days of psychiatric care	1.032	0.003	< .001	1.025, 1.039
Days of physical problems	1.067	0.001	< .001	1.065, 1.069
Age at first drug use	0.998	0.002	.210	0.995, 1.001
Days of primary drug use	0.996	0.001	.001	0.994, 0.998
Primary drug problem				
Alcohol^a^				
Cocaine	1.790	0.075	< .001	1.649, 1.942
Heroin	1.113	0.051	.020	1.017, 1.218
Marijuana	1.194	0.059	< .001	1.083, 1.316
Methamphetamine	1.113	0.072	.096	0.981, 1.264
Other	1.620	0.090	< .001	1.454, 1.806
Children younger than 18	1.008	0.004	.036	1.001, 1.016
Program modality				
Outpatient^a^				
Methadone	0.964	0.116	.763	0.761, 1.221
Residential	1.606	0.068	< .001	1.479, 1.744

Note: ER, emergency room; IRR, incidence rate ratio. IRRs can be interpreted as the estimated rate ratio for a 1-unit increase in the independent variable, given the other variables are held constant in the model. For example, if days of physical problems increased by 1 point, the ratio for number of ER visits would be expected to increase by a factor of IRR = 1.067, while holding all other variables in the model constant.

Wald chi-square tests with degrees of freedom (20) = 6693.30. The corresponding p-value is less than 0.0001.

^a^Reference category.

Moreover, individuals who reported more days of mental health counseling, psychiatric care, or physical problems reported more ER visits. Yet, individuals with a history of mental health issues and those who reported more drug use during the previous 30 days reported fewer ER visits. Compared with alcohol users, those who reported their primary drug to be cocaine, heroin, marijuana, or other substances were more likely to report ER use; this was also true for individuals in residential substance abuse treatment compared to those in outpatient treatment.

### Correlates of hospitalization

Similar patterns emerged in regard to hospitalizations (see Table 3). Compared with non-Hispanic Whites, Blacks and Hispanics spent fewer days in the hospital, as did men compared to women. Compared to alcohol users, individuals who reported heroin or methamphetamine as their primary drug of choice spent fewer days in the hospital, as did those who reported more drug use during the previous 30 days.

**Table 3:** Random effect negative binomial regression on days in hospital

	**IRR**	***SE***	***p***	**95% CI**
Race/ethnicity				
Non-Latino White^a^				
Black	0.862	0.043	.003	0.782, 0.951
Latino	0.913	0.039	.032	0.840, 0.992
Age	1.004	0.002	.038	1.000, 1.007
Male	0.904	0.034	.007	0.841, 0.972
Education	1.000	0.007	.968	0.987, 1.013
Homeless	1.400	0.055	< .001	1.297, 1.512
History of mental health issues	0.623	0.023	< .001	0.579, 0.671
Days of mental health counseling	1.022	0.004	< .001	1.013, 1.031
Days of psychiatric care	1.064	0.004	< .001	1.056, 1.071
Days of physical problems	1.074	0.002	< .001	1.071, 1.077
Age at first drug use	1.003	0.002	.258	0.998, 1.007
Days of primary drug use	0.994	0.002	< .001	0.991, 0.997
Primary drug problem				
Alcohol^a^				
Cocaine	1.695	0.099	< .001	1.512, 1.901
Heroin	0.791	0.052	< .001	0.695, 0.901
Marijuana	0.929	0.064	.281	0.812, 1.062
Methamphetamine	0.831	0.078	.047	0.692, 0.998
Other	1.222	0.100	.014	1.041, 1.434
Children younger than 18	1.008	0.006	.143	0.997, 1.020
Program modality				
Outpatient^a^				
Methadone	1.034	0.130	.789	0.809, 1.323
Residential	1.821	0.092	< .001	1.650, 2.010

Note: IRR, incidence rate ratio. IRRs can be interpreted as the estimated rate ratio for a 1-unit increase in the independent variable, given the other variables are held constant in the model. For example, if days of mental health counseling increased by 1 point, the ratio for number of ER visits would be expected to increase by a factor of IRR = 1.022, while holding all other variables in the model constant.

Wald chi-square with 20 degrees of freedom = 5313.21. The corresponding p-value is less than 0.0001.

^a^Reference category.

Similar to emergency room visits, other factors were significantly associated with more time spent in the hospital, including being older and homeless. Additionally, individuals who received more mental health counseling, more inpatient psychiatric care, and reported more physical health problems during the previous 30 days experienced more days of hospitalization. Those reporting cocaine or other drugs not listed as their primary substance of choice experienced more days of hospitalization compared to alcohol users. Finally, individuals in residential substance use treatment reported more days of hospitalization compared to those in outpatient treatment.

## Discussion

Several characteristics were associated with the likelihood of receiving ER services or being hospitalized among individuals entering substance abuse treatment. Our findings support previous work that noted increased use of the ER by people with increased need for mental health and substance use treatment services [3] and extend the knowledge base by highlighting specific features. In particular, individuals reporting co-occurring physical conditions, increased use of mental health services (i.e., counseling and psychiatric care), and receiving residential compared to outpatient treatment reported the highest risk of using the ER more often and staying longer in the hospital.

By considering these characteristics, substance use treatment providers can create screening tools that allow for early identification of and intervention for health-related risk factors that increase the likelihood that individuals will use the ER, be hospitalized, or both. Designing and implementing programs to decrease ER use among clients in substance abuse treatment can be accomplished through preventative measures and coordination of integrated primary and behavioral health care. An emphasis on identifiable and modifiable characteristics (such as mental wellness) is a critical component of this approach.

Our conclusion is supported by the work of other researchers [18], who determined that an integrated medical and substance abuse treatment program helped decrease patient use of the ER. Others have reached a similar conclusion [21]; they suggested that outpatient treatment for substance abuse disorders and depression can efficiently decrease hospitalization rates and costs. This may be accomplished through systematic delivery of contingency management treatment in community-based settings [21-23].

### Limitations

These findings highlight important characteristics that can help identify individuals in substance use treatment who are most likely to use hospital services; however, limitations must be noted. Although racially and ethnically diverse, individuals in this study represented only one county in California; characteristics associated with ER use and hospitalization among substance users in treatment may differ by region. The current analysis focused on ER visits and hospitalization 30 days prior to entering treatment. It may be that there is an association between an ER/hospital event and substance abuse treatment admission. As such, future research should not only include a longer time frame, but consider the examination of acute health events and their role in precipitating entry into substance abuse treatment.

## Conclusion

These robust findings, based on four years of data from the most populous county in the United States, are important because they suggest identifiable characteristics that can be targeted to reduce the overuse of hospital services by mainly racial and ethnic minority individuals in substance use treatment.

## Competing interests

The authors declare that they have no competing interests.

## Authors’ contributions

JC was responsible for conceptualization, writing, and revision of the manuscript. EG was responsible for the creation of statistical models, data analyses, writing, and revision of the manuscript. KM was responsible for writing and revision of the manuscript. TK was responsible for the creation of statistical models and assistance with data analyses. All authors read and approved the final manuscript.
